# The impact of fiscal pressure on education expenditure: Evidence from China

**DOI:** 10.1371/journal.pone.0327484

**Published:** 2025-06-27

**Authors:** Jie Yan, Yashu Qin, Xiaoyun Gong

**Affiliations:** 1 School of Public Finance and Taxation, Southwestern University of Finance and Economics, Chengdu, Sichuan, China; 2 School of Economics, Foshan University, Foshan, Guangdong, China; Southern Illinois University Carbondale, UNITED STATES OF AMERICA

## Abstract

Adjusting the tax distribution relationship among governments at all levels in the reform of the fiscal and taxation system will inevitably trigger changes in local government fiscal revenue. Will the fiscal pressures accompanying such changes have a significant impact on the expenditure decisions of local governments? Drawing on the 2002 Income Tax Sharing Reform in China as a quasi-natural experiment, we apply an intensity difference-in-difference methodology to evaluate how fiscal pressure influence county-level education provision. The empirical evidence indicates that counties most exposed to the reform experienced a marked reduction in the proportion of fiscal expenditure devoted to education, with the impact exhibiting a lagged and persistent pattern. The heterogeneity analysis reveals that fiscal pressure has a more pronounced negative impact on education expenditure in counties with developed economies, lower pre-reform education expenditure ratios, and outflow of transfer payments, while special transfer payments can better alleviate this negative effect. In addition, we also found that county governments will give priority to cutting education expenditure after suffering a fiscal shock, and intergovernmental competition further amplifies the adverse effect of fiscal pressure on county education expenditure.The analysis and conclusions of this article help to explain the reasons for insufficient education expenditure at the county level across China, thereby providing effective suggestions for the local government’s fiscal expenditure decision-making choices under fiscal pressure, and also providing important inspiration for advancing modern fiscal and tax system reforms.

## Introduction

Education is a vital component of national strategy, and the functioning of all education systems depends on fiscal support. Public finance, as a policy instrument and material foundation for the government to fulfill its functions, and is key to underpinning educational transformation and progress. The Education 2030 Framework for Action, launched in 2015, proposes that “allocating at least 4% to 6% of gross domestic product to education and/or allocating at least 15% to 20% of public expenditure to education” as international benchmarks for education expenditure [1]. Nevertheless, the latest data from the UNESCO Global Education Monitoring Report 2024 shows, globally, the median percentage of GDP allocated to public education expenditure decreased from 4.4% to 4% between 2015 and 2022,while the proportion of public expenditure allocated to education decreased from 13.2% to 12.6%.In terms of dual international benchmarks for education expenditure, 59 out of 171 countries failed to meet the two targets, comprising mainly the majority of lower-middle-income and low-income countries [2]. These data reflect that the issue of inadequate fiscal support for education is common around the world.

In 1993, China introduced its Outline for Education Reform and Development, which for the first time set a clear objective “ national fiscal expenditure on education should reach 4% of GDP by the end of the 20th century “. China’s Education Modernization 2035 promulgated in 2019 once again pointed out that “Consistently increasing education funding within the public budget each fiscal year is a crucial pillar in the pursuit of modernizing our educational system”. Subsequently policy documents issued by the Chinese government have repeatedly emphasized the need to ensure sufficient investment in education, and have actively implemented various measures to foster the realization of this goals. Despite prolonged efforts, the anticipated outcomes have not been realized, and akin to numerous emerging nations, China’s educational expenditure stays moderately low. Although China has finally achieved the goal of “education expenditure accounting for 4% of fiscal expenditure” at the national level for the first time nearly 20 years after the policy target was proposed, this macro average masks the structural imbalance among local levels. The proportion of counties that actually meet this expenditure standard is still less than 55%, and the phenomenon of insufficient fiscal education expenditure is more prominent in counties.

Scholars have put forward various perspectives on why there has been a prolonged lack of adequate investment in China’s education funding, and their analyses have primarily applied fiscal decentralization and political promotion incentives theories to explain the phenomenon. Following the 1994 reform of the tax-sharing system, the central government has modified the distribution relationship through a series of reform measures to achieve its dominant position in fiscal revenue. While fiscal authority have been continuously shifting upwards, local governments have found themselves shouldering the primary financial responsibility for various critical areas, including basic education, healthcare and social security, etc. [3]. The growing fiscal imbalance caused by the mismatch between revenue and expenditure responsibilities has significantly weakened local governments’ capacity and incentives to deliver essential non-economic public services [4,5]. Thus, scholars argue that fiscal decentralization in China has failed to enhance local welfare [6–8]. In particular, influenced by mechanisms for the promotion of officials, economic growth and fiscal revenue increase have become the main objectives for assessing officials [9], and as the competition among governments around the target intensifies, local governments are compelled to establish expenditure patterns emphasizing economic growth while overlooking social welfare concerns [10–12], leading to capital investment expenditures crowding out fiscal resources that would otherwise be allocated to public service provision [13–15].In this context, education as a typical public good characterized by non-profit, strong spillover, slow promotion of human capital, and insignificant short-term economic growth effects, the local administration’s prioritized expenditure will necessarily omit the allocation to educational funding, thereby causing a considerable decline in educational resources.

The above researches provide good theoretical explanations for China’s insufficient education expenditure. However, an important limitation of these studies is that they all ignore the potential impact of changes in fiscal constraints on the adjustment of government expenditure structure. In fact, when local fiscal encounter external shocks and budget space is tightened, local governments are no longer faced with the question of whether they are willing to invest in education, but rather whether or not they are still able to maintain fiscal support for education. Accordingly, numerous studies have examined how local governments respond to fiscal constraints. For instance, Nelson and Balu (2014) [16] pointed out in their empirical study of the US K-12 education system that local governments typically respond to fiscal austerity by reducing human resource costs. Castro (2017) [17] analyzes EU panel data to show that fiscal consolidation leads to significant cuts in education, health, and social protection expenditure, indicating fiscal pressure reshapes expenditure structures and undermines basic public services. Similarly, Justino et al. (2023) [18] take the United Kingdom as an example, revealing that it made large-scale cuts in public services and welfare expenditure after the fiscal crisis to curb the rise in public debt. In China, Xi and Huang (2020) [19] find that under fiscal constraints, local governments often sacrifice social security expenditure to meet the mandated 4% education funding ratio target. Moreover, eliminating the agricultural tax weakened local governments’ stable income streams, counties more severely impacted by this reform experienced a significant drop in the share of education expenditure, accompanied by a growing fiscal bias toward infrastructure investment [20]. All these suggests that fiscal constraints have had a substantial impact on local fiscal expenditure arrangements. In most cases, local governments tend to adopt a strategy of cutting expenditures [21]. Therefore, it is necessary to incorporate fiscal constraints into the research scope of educational fiscal behavior.

Although the current literature has focused on how fiscal pressure influence the government’s decision-making on expenditure, there are still some shortcomings in these studies. First, the existing research results pay more attention on the long-term or cyclical structural changes in overall local government expenditures triggered by fiscal institutional arrangements and political factors, ignoring the independent impact of exogenous shock factors. Therefore, there is a lack of discussion on the adaptive adjustments made by local governments in expenditures to cope with crises under fiscal pressure. Second, existing research on fiscal pressure has mainly focused on higher-tier governments, overlooking the more significant fiscal pressure faced at county and sub-county levels. Compared with governments at other levels, county-level finances face stronger budgetary constraints and more direct expenditure responsibilities, and their behavioral characteristics and coping strategies are significantly different, which urgently requires empirical support at the micro level. Third, in the identification of fiscal pressure, the indicators used are difficult to avoid endogeneity problems caused by reverse causality or omitted variables, which affects the validity of causal inference. Fourth, current research on fiscal pressure focuses on its overall effect, lacking a separate discussion on specific expenditure areas, especially education expenditure, a typical quasi-public goods investment.

This article employs county-level data from China spanning 2000–2007 and utilizes the reform in 2002 as a quasi-natural experiment to empirically examine the effects of fiscal pressure on public education expenditure. In comparison to previous studies, the efforts undertaken in this paper are: First, distinguishing from previous analytical frameworks, this paper utilizes the exogenous fiscal shock triggered by the 2002 Income Tax Sharing Reform to not only explore the immediate response of county governments in response to the short-term crisis, but also effectively alleviate the identification bias brought about by the endogeneity of the dependent variable in the traditional measure of fiscal pressure. Second, the analysis based on county-level data, focusing on the weakest fiscal capacity and the decision-making body of education expenditure arrangements, can more realistically reveal the fiscal vulnerability and dependence shown by county governments under fiscal pressure, and help to make up for the lack of attention paid to county government expenditure behavior in existing studies. Third, this research not only verifies the impact of fiscal pressure on county-level education expenditures, but also strengthens the credibility of its findings through rigorous robustness checks, on the basis of which it carries out the analysis of heterogeneity and the identification of mechanisms. The conclusions contribute to the literature on local government education expenditure behavior and offer empirical support for the institutional design of fiscal reform and the optimization of government expenditure structure.

## Institutional background

### Local education expenditure in China

**Fig 1:**
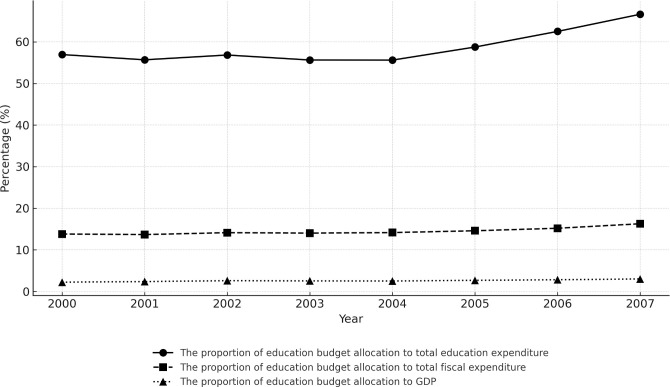
Structural changes in China’s fiscal education expenditure from 2000 to 2007. Note: These dates are sourced from the China Statistical Yearbook.

Adequacy of financial resources is an important guarantee for achieving high-quality education [22]. The Dakar Framework for Action also emphasizes that governments should play a major role in providing funding for education for all, especially allocating sufficient funds to all sectors of basic education [23]. Since the 1980s, China’s education financing method has gradually shifted to a diversified model with government investment as the main source. The purpose of designing a diversified financing mechanism is to reduce the government’s fiscal burden and enhance the effectiveness of educational resource distribution. But in practice, education funding is still mainly borne by the government, especially for basic education, which relies heavily on local government supply [24]. Fig 1 illustrates the evolution of China’s education expenditure structure from 2000 to 2007. The statistics indicate that government budget allocations have been on the rise annually, now constituting over 60% of the overall funding for education.

**Fig 2:**
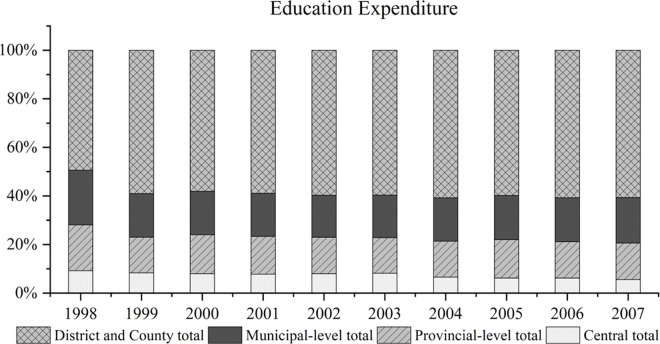
Proportion of education expenditure borne by different levels of government (1998-2007). Note: Collated and calculated according to Finance Statistics of Prefectures, Cities, and Counties in China.

Subsequent to the 1994 reform of the tax-sharing system, with the decentralization of expenditures from the central to the provincial level, the provincial governments have also shifted the focus of public service expenditures to lower-level governments, and education expenditure was mainly borne by the county government [25,26]. Fig 2 illustrates the distribution of education expenditure across central, provincial, municipal and county levels from 1998 to 2007. Among them, county governments have made the greatest contribution to education expenditure, with the proportion remaining above 60% and showing an increasing trend. The above analysis shows that ensuring sufficient fiscal investment in education is not only a necessity for fostering local educational advancement, but also a crucial obligation for county governments.

However, under the education management system of “responsible by local governments, but mainly by counties”, local governments have consistently underfunded education, lagging behind the speed of economic growth and the expansion of education scale. The scale of educational fiscal expenditure is mainly limited by the fiscal resources that the localities themselves possess. Due to various factors such as regional economic differentiation and differences in resource endowments, the ability of different levels of government to organize fiscal revenues varies greatly from region to region. In particular, for most county governments, tax revenues they can obtain is relatively limited because of the lack of economic support, and it is difficult to meet the educational needs of their jurisdictions with their own fiscal resource [27]. Despite the central government’s continued expansion of transfer payments to less developed areas in recent years, the role of transfer payments on addressing the shortfall in educational funding is also relatively limited [28–30], the fiscal difficulties of local basic education have still not been effectively resolved.

### Background of China’s income tax sharing reform

The intergovernmental distribution of fiscal revenue constitutes a core element of the fiscal framework. The 1994 tax-sharing reform classified taxes into three categories: central, local, and shared taxes. As a typical example of local taxation, the corporate income tax is allocated based on corporation affiliation, with all revenues fully retained by the local governments where the corporations are registered. However, this method of tax distribution has led to excessive competition and protectionist behavior among local governments, exacerbated the economic and financial gaps between regions, and hindered the coordinated development of regional economies.

In order to maintain fiscal stability and promote balanced regional development, China has embarked on a new phase of reforms aimed at its fiscal and taxation systems, readjusted the vertical distribution relationship among governments. On December 31, 2001, the government enacted the “Income Tax Sharing Reform Program,” effective January 1, 2002. Following the reforms, the corporate income tax, which used to be entirely owned by local governments, is now distributed proportionately between both the central and local authorities. Taking the actual local income tax revenue in 2001 as the base for each region, the central government implements incremental sharing while ensuring that each region receives the base amount, the scope and proportion of income tax sharing are unified nationwide. In 2002, the revenue split between central and local governments was an even 50−50. However, beginning in 2003, the local share was reduced to 40%, with the central government retaining the flexibility to modify the distribution percentages in subsequent years depending on circumstances. Simultaneously, the central government will allocate all additional revenue to general transfer payments, particularly targeting the central and western regions, to rectify the fiscal imbalances among various regions and to guarantee that local governments can furnish essential public services.

The existing literature regarding the implications of the Income Tax Sharing Reform primarily examines how the reform has influenced tax collection practices and the management strategies adopted by tax agencies [31], and the resulting impact on business operations or decision-making of enterprises [32,33], but scholars have predominantly neglected the economic challenges encountered by local authorities post-reform and the consequent alterations in their spending patterns.

The corporate income tax serves as a vital sources of tax revenue for governments at all level. Especially after the 1994 reform, governments at all levels have taken various measures to strengthen the development of tax sources, and as a result, corporate income tax revenue has grown rapidly. The proportion of local corporate income tax in general public budget revenue increased from 13.76% in 1994 to 21.6% in 2001, at the county level, this proportion was 19.08% in 2001.However, following the reform, local governments retained only residual access to corporate income tax, which had become a shared tax. The income tax revenue will be greatly reduced, and these losses will inevitably cause increased fiscal pressure in most regions.

**Fig 3:**
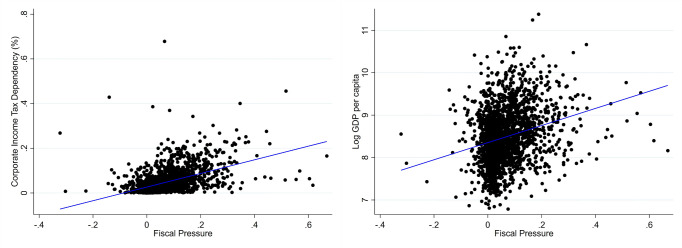
Scatter plot of fiscal pressure and other variables.

To provide a more intuitive understanding of the fiscal pressure indicator used in this study, we construct two scatter plots using 2002 data, showing its correlation with major determinants. As shown on the left side of Fig 3, a strong positive link exists between fiscal pressure and corporate tax share, suggesting that counties that are more dependent on corporate income tax in the structure of tax sources are subject to greater fiscal shocks in the 2002 reform, thus showing stronger fiscal pressure. The figure on the right further shows a similar positive association between fiscal pressure and GDP per capita, indicating that economically developed counties have stronger income tax bases, so they are more significantly impacted. These results not only fit the characteristics that increasing the tax sharing ratio is unfavorable to redistribution in high-tax counties, but also verify the rationality and effectiveness of the fiscal pressure variable from a visual level.

The preceding analysis reveals that the 2002 Income Tax Sharing Reform has put county governments under completely different constraints, and the passive centralization of tax revenues has exogenously weakened the fiscal autonomy of the county governments. Does the fiscal pressure caused by this exogenous shock restructure their expenditures, and in particular, does it have an impact on investment in education? If so, what are the fiscal decision-making mechanisms behind this behavioral adjustment? What kind of heterogeneity will this impact present under different conditions? In addition, can the changes in transfer payment policies accompanying the reform really produce a compensatory effect? These are the key issues that this article needs to explore.

## Empirical research design

### Model setting

This study employs the 2002 Income Tax Sharing Reform, a nationwide policy implemented concurrently across all counties, as a policy experiment to measure changes in county-level fiscal pressures, and chooses the intensity difference-in-difference model to examine the potential expenditure behaviors and characteristics of county-level governments in response to fiscal pressures. The baseline regression model of this paper is as follows:


$${Eduexp}_{it}=\beta_{0}+\beta_{1}{Pressure}_{i}*{post}_{t}+\beta_{2}X_{it}+\alpha_{t}+\gamma_{i}+\varepsilon_{it}$$ (1)

Where i signifies the county and t indicates the year. The variable ${Eduexp}_{it}$ represents the size of education expenditure in the county. ${Pressure}_{i}$ is our measure of the fiscal pressure on county i; ${post}_{t}$ is a dummy variable for dividing policy time points, if the time is in 2002 or later, it is 1; if it is before 2002, it is 0. $X_{it}$ encompasses all control variables. $\alpha_{t}$ is a year fixed effect, $\gamma_{i}$ refers to county-specific effect for county i, $\varepsilon_{it}$ is the random disturbance term. $\beta_{1}$ is the parameter to be estimated and the estimated coefficient is expected to be negative, then it suggests that counties with stronger fiscal shocks will reduce their fiscal expenditure on public education more substantially after this reform.

### Variable design

The dependent variable is county education expenditures. Drawing on prior literature, this study measures the scale of county-level fiscal education supply by calculating education expenditure as a share of total public budget expenditures [14]. A greater value of this proportion signifies an increased investment in education within the county.

Key explanatory variable. Measured by the interaction term between the shock to county-level fiscal revenue caused by the 2002 reform and the dummy variable for the reform year. Although all counties’ revenues were hit by the policy at the same time, the extent of the fiscal loss and the response to the reform varied across counties because of significant regional differences in the contribution of corporate income tax revenues to county fiscal revenues. Referring to Chen (2016) [34], the fiscal shock induced by the 2002 reform is measured by the change in the ratio of county-level corporate income tax revenue to total fiscal revenue, defined as the difference between the average ratio in the pre-reform period (2000–2001) and the post-reform period (2002–2007), the formula for the specific measure is as follows:


$${Pressure}_{i}=\frac{{corptax}_{i,2000-2001}}{{suminc}_{i,2000-2001}}-\frac{{corptax}_{i,2002-2007}}{{suminc}_{i,2002-2007}}$$ (2)

In the formula (2) provided about,${corptax}_{i,2000-2001}$ represents corporate income tax revenue before this tax reform, ${suminc}_{i,2000-2001}$ shows pre-reform general public budget revenue; ${corptax}_{i,2002-2007}$ represents corporate income tax revenue after this tax reform, ${suminc}_{i,2002-2007}$ reflects post-reform public budget revenue.

Control variables. To maintain comparable external conditions for both the experimental and comparison groups, a series of variables related to economic structure and society, and demography were included in the regression model. Among them, the GDP per capita represents the regional economic level, the percentage of the secondary sector within the GDP reflects the regional economic structure, the total population indicates the population distribution, the employed population describes the county’s employment activity, and the scale of education at the county level is measured by the number of secondary school students currently enrolled, and the size of county fiscal expressed in terms of fiscal expenditures per capita. To mitigate the skewness of variable distributions and enhance the stability of the estimation results, some control variables have been transformed using the natural logarithm.

### Data and descriptive statistics

The relevant data on fiscal revenues and expenditures used in this paper come from the Finance Statistics of Prefectures, Cities, and Counties in China, and the financial statistical yearbooks of the respective provinces. Control variables and other data from the China County (City) Socio-Economic Statistical Yearbook and provincial or municipal statistical yearbooks. In light of the significant changes in fiscal classifications introduced by China’s 2007 reform, which altered the structure of revenue and expenditure items, this study confines the sample period to 2000–2007 to ensure the comparability and consistency of fiscal variables. After excluding counties with substantial missing data, a county-level panel datasets covering 2017 counties was constructed. Table 1 outlines the descriptive statistics for primary variables.

**Table 1:** Descriptive statistics of main variables.

Variable Name	Number of observations	Mean value	Standard deviation	Minimum value	Maximum value
Eduexp	16131	24.484	6.668	2.485	55.408
${Pressure}_{i}$	16136	0.066	0.085	−0.322	0.669
lnPgdp	16081	8.729	0.750	6.038	12.040
Ind2	16089	0.367	0.203	0.070	14.213
lnPop	16113	12.703	0.944	7.900	15.653
lnEmp	16094	9.728	0.955	5.165	13.750
lnEdusize	16078	9.834	1.199	0.000	11.992
lnFis	16071	0.033	0.016	0.016	0.529

**Fig 4:**
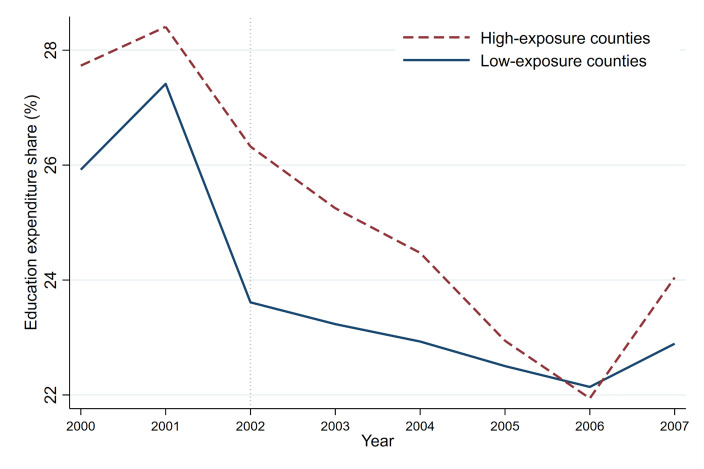
Trends in education expenditure share by fiscal pressure group (2000-2007).

To better illustrate how education expenditure has evolved in counties affected by reform to varying degrees, we categorize the sample into High-exposure and Low-exposure groups using the median value of our fiscal pressure index. High-exposure counties refer to those counties that are more likely to suffer revenue losses due to tax redistribution caused by reforms. Then we illustrate the evolving pattern of the proportion of educational expenditure within overall fiscal expenditure across the two county groups between 2000 and 2007, as depicted in Fig 4.

It can be observed that, prior to the reform, the share of education expenditure in high-exposure counties was noticeably higher than that in low-exposure counties. However, after the 2002 reform was implemented, the share of education expenditure in both groups of counties declined significantly, with high-exposure counties experiencing a larger decline and a steeper downward trend. By 2006, the share of education expenditure in high-exposure counties fell below that of low-exposure counties for the first time. Although there was a slight rebound in 2007, the gap between the two groups persisted. The changing trend presented in this figure is consistent with the empirical results below, which intuitively demonstrates the adverse effect of fiscal pressure on education expenditure, and is particularly evident in counties that are more affected by fiscal shocks.

## Empirical results

### Baseline regression

Table 2 displays the baseline regression outcomes. Column (1) includes only year and county-level fixed effects, while column (2) incorporates additional control variables. Then robust and county-clustered standard errors are included in columns (3) and (4), respectively. The cross-multiplier coefficients for fiscal shocks display a negative trend and achieve 1% significance in all evaluated models. This indicates that counties that are more affected by the Income Tax Sharing Reform have a lower proportion of education expenditure, that is, the baseline regression result preliminarily confirms that when fiscal pressure increases, county governments will adopt a pressure-relief strategy by cutting education expenditures.

**Table 2:** Baseline regression results for the effect of fiscal pressure on education expenditure.

Variables	(1)	(2)	(3)	(4)
${Pressure}_{i}*{post}_{t}$	-9.193^***^	−8.126^***^	−8.126^***^	−8.126^***^
	(0.875)	(0.878)	(1.780)	(1.780)
Control Var	No	Yes	Yes	Yes
County Fixed	Yes	Yes	Yes	Yes
Year Fixed	Yes	Yes	Yes	Yes
Observations	16131	15965	15965	15965
R-squared	0.200	0.212	0.212	0.212

Note: Standard errors in parentheses.

*** p < 0.01,

** p < 0.05,

* p < 0.1. The t-test values in parentheses. Same as below.

To further explore the temporal dynamics of how fiscal pressure affects education expenditure, we adopt the event study method and creates the fiscal pressure and relative year interaction term within the intensity DID model. Table 3 presents the regression analysis outcomes. The negative impact of fiscal pressure did not appear immediately in the year of policy implementation (${post}_{0}$for 2002), but it showed a significant negative effect from the second year and reached its peak in the third and fourth years. Although the effect weakened in the fifth year, it still remained significant. These results suggest that fiscal pressure exerts a delayed and lasting effect on education expenditure, which cannot be fully mitigated in the short run.

**Table 3:** Dynamic effects of fiscal pressure on education expenditure.

Variables	(1)
${Pressure}_{i}*{post}_{0}$	-2.761
	(2.003)
${Pressure}_{i}*{post}_{1}$	-5.145***
	(1.952)
${Pressure}_{i}*{post}_{2}$	-6.414***
	(2.039)
${Pressure}_{i}*{post}_{3}$	-10.05***
	(2.094)
${Pressure}_{i}*{post}_{4}$	-12.09***
	(2.152)
${Pressure}_{i}*{post}_{5}$	-5.476***
	(2.020)

Note: Control variables are consistent with the baseline regression.

### Robustness tests

To verify the baseline regression’s reliability, we conduct a series of robustness checks.

**Fig 5:**
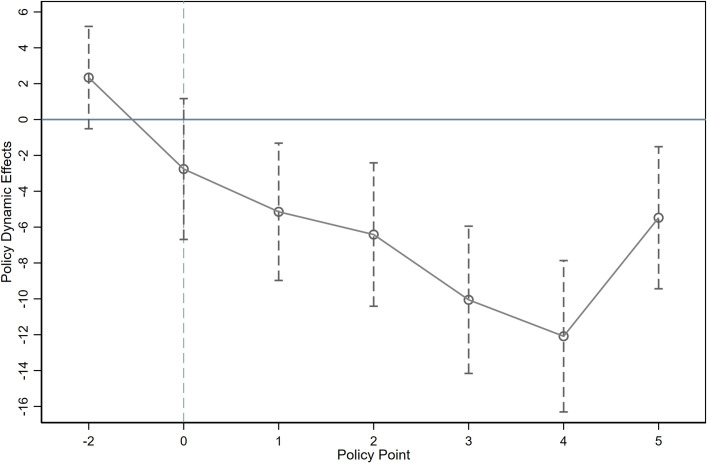
Parallel trend test.

Parallel trend test. A fundamental condition for applying the DID approach is that there are no substantial disparities between the experimental and comparison groups prior to the policy’s introduction. Applied to this study, it requires that county-level education expenditure followed a similar trend across groups prior to the 2002 reform. Drawing on the common practice in the existing literature, this study refines Post into time dummies for each year within the study period, then cross-multiplies this series of year dummy variables with pressure and adds them to Eq. 1 for testing. Fig 5 reports this result. The findings show that prior to the 2002 reform, none of the coefficients on the interaction terms of the fiscal shocks are significant, indicating that counties affected by different fiscal shocks have no significant differences in their education expenditures prior to the reform, aligning with the parallel trend assumption. In addition, although the marginal effect of fiscal pressure at t=−2 is positive and exhibits a certain visual deviation in the figure, the estimated value is statistically insignificant (p = 0.108) and the 95% confidence interval contains zero. Given the inherent volatility and structural heterogeneity of county fiscal data, we consider this deviation to be somewhat acceptable and does not affect support for the parallel trend hypothesis.

**Fig 6:**
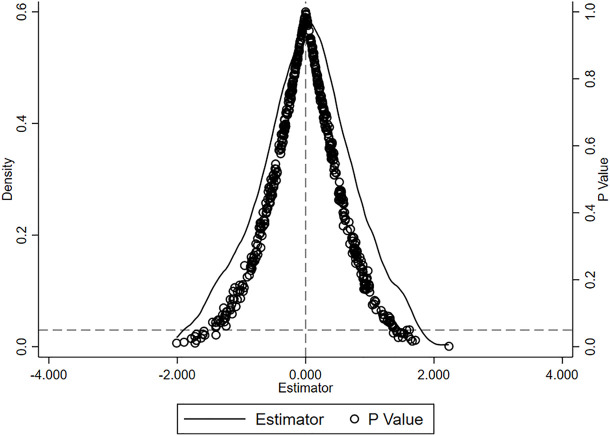
Placebo test.

Placebo test. To account for potential confounding factors and assess the reliability of the baseline results, this study conducts a placebo test as a robustness check. Specifically, we perform spurious estimation of the interaction term for randomly generated fiscal shocks and repeat it 500 times. The results are shown in Fig 6. The mean values of the estimated coefficients obtained are clustered around 0 and the P values of most estimators are greater than 0.1, which is significantly different from the baseline regression results. This shows that the negative effect of fiscal pressure generated by the 2002 reform on county-level education expenditure is not due to random factors.

Replace the measure of education expenditure. Firstly, instead of the previous dependent variable, we use the ratio of education expenditures to GDP, which is also one of the international benchmark indicators for measuring a country or region’s investment in education. Second, we also perform a robustness test using the natural logarithmic of per-student education expenditure as the dependent variable. This measure is derived by dividing total education expenditures by the enrollment figures for standard primary and secondary schools. After controlling for the same control variables as in the baseline regression, the above two variables reflecting the status of education expenditure are substituted for the original explained variables and put into the model regression. Table 4’s columns (1) and (2) depict the regression outcomes. Following adjustments to the variable measurements, the adverse effect of fiscal pressure on education expenditure remains statistically significant at the 5% and 1% confidence intervals, respectively, consistent with the findings of the baseline regression. In addition, the model using per-student education expenditure as the dependent variable indicates that fiscal pressure significantly compromises the allocation of educational resources at the individual level. Notably, this negative effect is more substantial than the reductions observed in macro-level budgetary allocations.

**Table 4:** Robustness tests results.

Variables	(1)	(2)	(3)	(4)	(5)
${Pressure}_{i}*{post}_{t}$	-0.533**	−0.195***	−1.646***	−5.975***	−7.902***
	(0.247)	(0.051)	(0.172)	(1.774)	(1.840)
Control Var	Yes	Yes	Yes	Yes	Yes
County Fixed	Yes	Yes	Yes	Yes	Yes
Year Fixed	Yes	Yes	Yes	Yes	Yes
Observations	15965	15970	15965	9976	14810
R-squared	0.450	0.592	0.225	0.143	0.206

Note: Clustering robust standard errors at the county level are in parentheses. Same as below.

Redefine the measurement of fiscal pressure. According to the requirements of the 2002 reform, the increased revenue from income tax sharing by the central government will be used entirely for general transfer payments to localities, and this measure will significantly influence county-level fiscal allocations. To precisely capture the financial pressure imposed by the reform, referring to Xu et al. (2020) [35], the general transfer payments are now included in the reconstruction of the fiscal pressure indicators. The estimation in column (3) of Table 4 confirms that the core interaction term retains a significantly negative effect at the 1% level. Nevertheless, the coefficient estimates decrease significantly from −8.13 in the original baseline model to −1.65. This result not only reinforce the robustness of the baseline estimates, but also provides strong evidence that central transfer payments greatly compensate for the losses caused by county governments due to the reform, effectively alleviates fiscal shocks’ negative effects on education expenditures, with the revised model better capturing their true impact.

Exclusion of other policy. During the period of this study, China implemented other reform measures that may have had an impact on county finances. For example, agricultural tax was completely abolished nationwide in 2005.After the abolition of this tax, the fiscal tax sources of county-level governments were once hit again, and their fiscal revenue could only rely on increasing business taxes and central transfer payments, thus making fiscal difficulties even more pronounced. In order to eliminate the effects of agricultural tax reform, we excluded the county samples from 2005 and later for regression. In 2004, China initiated a VAT reform pilot in three northeastern provinces, shifting from a production-based to a consumption-based system to alleviate enterprise tax burdens. But on the other hand, while the burden on enterprises is reduced, local government tax revenue is reduced and fiscal pressure is increased. To avoid any potential interference stemming from this reform, the samples from the three northeastern provinces have been excluded prior to the regression analysis. Columns (3) and (4) of Table 4 present the regression results after excluding the effects of the two aforementioned reforms. The results suggest that the interaction term capturing the fiscal shock continues to exhibit a significantly negative effect. These two reforms did not substantially alter the estimation outcomes of the baseline model.

### Heterogeneity analysis

The extent to which counties are affected by the 2002 reform may vary depending on their stage of economic development, so the responses of county governments to cope with the pressure will also be different. In order to verify the regional heterogeneity of fiscal pressure affecting education expenditure, the sample is segmented into two categories based on per capita GDP levels: High-GDP and Low-GDP counties. Table 5, columns (1) and (2) present estimation results indicating insignificant impact coefficients for economically disadvantaged counties, while the impact coefficients of economically developed counties are significantly negative. The reason for this is that the better economically developed, the greater the contribution of corporate income tax to local tax revenue, the more tax revenue will be adjusted after the reform. This result shows that when the government’s revenue structure is overly dependent on a single tax source, it is more sensitive to policy shocks and is more likely to cut education and other livelihood expenditures. Instead of demonstrating a strong capacity to secure public services under specific fiscal system changes, economically developed counties are more likely to reveal fiscal vulnerabilities.

**Table 5:** Heterogeneity analysis results.

Variables	High-GDP	Low-GDP	High-Edu	Low-Edu	Out	In	High-Gt	Low-Gt
	(1)	(2)	(3)	(4)	(5)	(6)	(7)	(8)
${Pressure}_{i}*{post}_{t}$	-7.136***	0.230	−1.104	−8.729***	8.463***	−2.884	−7.513***	−6.419**
	(2.691)	(1.650)	(2.199)	(2.179)	(2.569)	(2.358)	(2.474)	(2.738)
Control Var	Yes	Yes	Yes	Yes	Yes	Yes	Yes	Yes
County Fixed	Yes	Yes	Yes	Yes	Yes	Yes	Yes	Yes
Year Fixed	Yes	Yes	Yes	Yes	Yes	Yes	Yes	Yes
Observations	7486	8479	4446	11519	7506	8459	9058	6907
R-squared	0.323	0.111	0.095	0.264	0.256	0.169	0.239	0.16

The heterogeneous impact of education expenditure levels. To explore how the fiscal shock affected counties with varying levels of education expenditure, we categorized the sample into two groups, depending on whether the proportion of education expenditure in GDP reached 4% before the reform. Columns (3) and (4) of Table 5 report the empirical results, which show that counties where education expenditure accounted for less than 4% of GDP before the reform had significantly decreased in education expenditure after the fiscal shock, while the interaction term coefficients for the fiscal shock are insignificant in counties where education expenditure exceeded 4%. The reason for this is that counties with higher education expenditures as a share of GDP are instead found in less economically developed areas. On the one hand, economically developed counties have larger GDPs, thereby complicating the quest to reach the 4% GDP education expenditure goal. On the other hand, for these counties, investment in education is not a fiscal priority, and they tend to prefer investing limited fiscal resources in areas that can directly promote GDP growth. The expenditure preference of “economy over people’s livelihoods” may become more pronounced when fiscal pressures intensify.

In the last dimension, we consider the heterogeneous impact of central transfers. The reform’s policy framework noted that the central government would use the revenue gains from tax sharing to expand fiscal transfers to central and western regions, in an effort to reduce fiscal imbalances across regions. In this way, the fiscal burden for counties receiving higher levels of transfer payments will necessarily ease to a degree, so the ratio of education expenditure may not be affected. To test this claim, we refer to Chen SX et al. (2017) [36] to verify this statement, and treat the counties that received no or few general transfers before the reform as transfer outflows, assigned a value of 1; and the rest as transfer inflows, assigned a value of 0. Columns (5) and (6) of Table 5 display the findings from the grouped regression analysis. The results show that counties experiencing outflows of transfer payments tend to cut back on their education expenditure when faced with the fiscal strain resulting from the reform. This suggests that the compensation mechanism, which focuses on supporting the central and western regions, has to some extent ignored the developed counties that have been hit harder by the reform, resulting in them having to cut non-rigid expenditures such as education to maintain fiscal balance after a sharp drop in fiscal resources. In contrast, the central and western regions have been cushioned from the negative impact of fiscal pressures on education expenditure by receiving more fiscal support. However, the fiscal stability they showed after the reform shock did not stem from their own improved fiscal capacity, but instead may have increased their dependence on transfers.

To identify which type of transfer mechanism is more effective in mitigating the adverse impact of fiscal pressure on education expenditure, we construct a transfer structure indicator, defined as the ratio of general transfers to earmarked transfers. Using the median ratio, we categorize the sample into high and low general transfer payment groups, and conduct subgroup regressions to assess whether the impact of fiscal pressure differs across transfer structures. As presented in columns (7) and (8) of Table 5, fiscal pressure significantly reduces education expenditure in both groups. Notably, the magnitude of the negative effect is larger in counties with a higher share of general transfers. This finding suggests that general transfers, as an important supplement to county-level fiscal funds, have enhanced fiscal autonomy, but in the absence of clear guidance on their use, they have not effectively alleviated the fiscal pressure to cut education investment. On the contrary, they may have exacerbated expenditure bias due to the expansion of discretionary space in the use of funds.

### Further analysis

We try to explore the internal mechanism of fiscal pressure affecting county-level government education expenditure from the perspective of fiscal expenditure structure adjustment. We select expenditure items in different functional areas such as social security expenditure, administrative expenditure, public security expenditure, and capital construction expenditure to identify the allocation tendency of fiscal resources under fiscal pressure. Empirical estimates are provided in columns (1) to (4) of Table 6, where county governments exhibit significant differences in expenditure adjustments: the 2002 reform had a slight but statistically significant negative impact on social security expenditures, no significant impact on administrative expenditures, but a significant increase in both public security and capital construction expenditures. The above results show that fiscal pressure did not lead to a general reduction in all types of fiscal expenditure, but rather prioritized rigid expenditures to ensure administrative operations and politically sensitive expenditures, and tilted towards increasing capital expenditures with growth effect (such as capital construction expenditure).In contrast, education expenditure, a type of social expenditure with higher elasticity and lower short-term returns, is more likely to be the first to be compressed in the event of fiscal tightening due to the lack of hard constraints and special guarantees.

**Table 6:** Further analysis results.

Variables	Secuexp	Admexp	PubSecexp	CapConexp	Eduexp
	(1)	(2)	(3)	(4)	(5)
${Pressure}_{i}*{post}_{t}$	-0.011**	0.006	0.010***	0.073***	−21.292***
	(0.005)	(0.008)	(0.003)	(0.023)	(3.160)
${Pressure}_{i}*{post}_{t}*compete$					0.194***
					(0.035)
Control Var	Yes	Yes	Yes	Yes	Yes
County Fixed	Yes	Yes	Yes	Yes	Yes
Year Fixed	Yes	Yes	Yes	Yes	Yes
Observations	15970	13964	13964	9478	15965
R-squared	0.69	0.115	0.088	0.046	0.217

In addition, considering that county governments are not completely autonomous in their expenditure arrangements and their behavior is also affected by external competitive pressure, we further introduce the intergovernmental competition variable to test its regulatory effect on education expenditure adjustments under fiscal pressure. The method assesses competition levels by counting the number of districts and counties within the same municipality, with a higher number of districts and counties indicating a higher degree of intergovernmental competition. Construct a triple difference term of the interaction between the competition index and the fiscal shock and add it to the regression equation. Table 6’s Column (5) displays the regression outcomes, with the triple-difference coefficient strongly positive at 1%. This suggests that heightened competition between local governments amplifies the negative impact of fiscal constraints on education expenditure. This means that in the context of increasingly fierce inter-governmental competition, fiscal games oriented towards economic performance may intensify the squeeze on people’s livelihood expenditures, further compressing the already fragile fiscal education expenditures.

## Conclusions and recommendations

Drawing on the 2002 Income Tax Sharing Reform as a policy experiment and panel data from Chinese counties, this study analyzes how fiscal pressure influences public education provision by county governments. The research results indicate: First, the 2002 reform placed considerable financial pressure on local governments, leading to a noticeable decline in education expenditure at the county level. Areas that bore the brunt of these fiscal changes saw the most dramatic cuts to their education budgets, with effects that were not immediate but long-lasting. Second, heterogeneity analysis shows that fiscal pressure impacts developed economies, pre-reform insufficient education expenditure relative to GDP, and counties with transfer payments outflows more severely. Third, government transfers have helped ease the negative impact of fiscal pressure somewhat, with special transfer payments proving more effective than general transfer payments. Fourth, mechanism analysis shows that the reduction in education expenditure is not the result of universal fiscal compression, but the selective expenditure adjustment behavior of county-level governments under fiscal constraints. Fifth, further analysis found that intergovernmental competition significantly exacerbated the negative effect of fiscal pressure on county-level education expenditure.

In light of these findings, several policy recommendations are proposed to enhance the design of fiscal institutions and ensure stable education expenditure in the future.

First, establish an education expenditure guarantee mechanism. On the one hand, set and strictly implement the minimum education expenditure standard at the county level, and provide a bottom-line support through the transfer payment mechanism to ensure that basic education expenditure is not compressed due to financial constraints; on the other hand, strengthen the rigid constraints on education expenditure, clarify its priority in budget implementation and adjustment, and effectively prevent expenditure reduction inertia through institutional constraints to avoid long-term slump in education expenditure due to persistent fiscal pressure.

Second, implement differentiated fiscal support strategies. Combine the county’s own fiscal resources, tax source structure characteristics, expenditure responsibility scale and other indicators to build a dynamic fiscal risk assessment system, identify high-risk counties that are more severely impacted by the reform, pay attention to implicit fiscal fragility, and provide graded and classified support based on this to enhance their fiscal resilience and ability to resist risks. Furthermore, for counties with weak fiscal capacity, greater emphasis should be placed on preferential fiscal policies and dedicated development funds. Efforts should be made to support these regions in strengthening their own revenue bases through industrial development and capacity building, thereby improving their ability to sustain basic public service provision.

Third, optimize the transfer payment system. This can be achieved by establishing a policy compensation mechanism aligned with the impact of the reform, providing phased compensatory support to counties experiencing fiscal losses and net outflows of transfer payments, in order to enhance their fiscal stability. Meanwhile, maintaining steady growth in general transfers should be complemented by a greater allocation of special transfer payments to education, clarify the purpose of funds and assessment standards, and prevent funds from deviating from public service goals.

Fourth, reform the existing fiscal performance evaluation mechanism. Weaken the GDP-oriented competition model, increase the weight of social development indicators including the ratio of education expenditure and per student expenditure within the fiscal performance assessment framework, form fiscal incentives oriented towards public services, and actively promote the construction of cross-county fiscal coordination mechanisms. Avoid the problem of race to the bottom leading to the squeeze of education expenditures through institutional constraints and coordination.

## Supporting information

S1 File Data. (XLSX)
